# Septic Shock Precipitating a Pheochromocytoma Crisis: A Case of Sequential Acute Myocardial Infarction and Cardiogenic Shock

**DOI:** 10.7759/cureus.98433

**Published:** 2025-12-04

**Authors:** Xianggui Xiong, Jiajia Zheng, Hongchao Zhang, Min Liu, Ling Wang

**Affiliations:** 1 Department of Infectious Diseases, Huanghua People's Hospital, Huanghua, CHN

**Keywords:** acute myocardial infarction, cardiogenic shock, catecholamine cardiomyopathy, gastroenteritis, pheochromocytoma, septic shock

## Abstract

Pheochromocytomas are rare neuroendocrine tumors that can cause life-threatening cardiovascular complications due to excessive catecholamine secretion. We report an unusual case in which acute gastroenteritis served as the trigger for a severe pheochromocytoma crisis.

A 64-year-old woman with a history of an undiagnosed left adrenal mass presented with a two-day history of nausea, vomiting, and diarrhea. She rapidly deteriorated into septic shock, requiring vasopressor support and hydrocortisone. Subsequently, she developed an acute myocardial infarction and heart failure. During hospitalization, she exhibited dramatic blood pressure fluctuations (60-220/40-110 mmHg), with paroxysmal hypertensive crises accompanied by the classic triad of palpitations, headache, and profuse sweating. Laboratory studies revealed markedly elevated plasma and urinary catecholamine levels. Abdominal computed tomography (CT) confirmed a 3.3 cm left adrenal mass, leading to the diagnosis of pheochromocytoma.

This case underscores that common infections, such as acute gastroenteritis, can act as potent stressors to precipitate a pheochromocytoma crisis, resulting in a complex and life-threatening presentation including septic shock, cardiogenic shock, and acute myocardial infarction. Early recognition of this potential trigger and the underlying tumor is critical for guiding appropriate management and preventing catastrophic outcomes. Physicians should maintain a high index of suspicion for pheochromocytoma in patients with unexplained hemodynamic instability, even in the setting of a common infectious illness.

## Introduction

Pheochromocytomas are rare neuroendocrine tumors, with an estimated annual incidence of 2-8 cases per million, and are a classic cause of secondary hypertension [1]. The clinical presentation is highly variable, most commonly featuring episodic headaches, sweating, palpitations, and hypertension. The serious and potentially lethal cardiovascular complications of these tumors are attributed to the potent effects of secreted catecholamines [1]. Critically, various stressors can trigger a pheochromocytoma crisis, characterized by a massive, unregulated release of catecholamines leading to profound hemodynamic instability and end-organ damage. Herein, we report an exceptionally rare case that underscores the potency of such triggers, in which a common infection (acute gastroenteritis) precipitated septic shock, which in turn provoked a pheochromocytoma crisis manifesting as acute myocardial infarction and cardiogenic shock.

## Case presentation

A 64-year-old woman was admitted to our hospital (Day 1) with a two-day history of nausea, vomiting, and diarrhea. Her medical history was significant for recurrent episodes of dizziness and hypotension over the past two years, which had not been formally evaluated. She had no personal history of smoking or alcohol use, and no family history of hypertension.

The present illness commenced after the patient ingested leftover rice. She subsequently developed nausea, vomiting, diarrhea, dizziness, and fatigue. She initially sought treatment at a community hospital, where administration of ciprofloxacin and smectite alleviated the vomiting and diarrhea; however, the nausea persisted. A chest and abdominal computed tomography (CT) scan revealed mild pulmonary edema, inflammatory changes in the ileum, and a left adrenal mass. Laboratory studies demonstrated markedly elevated levels of white blood cell count (WBC), C-reactive protein (CRP), procalcitonin (PCT), troponin I, and B-type natriuretic peptide (BNP). Consequently, she was admitted to our institution with a preliminary diagnosis of acute gastroenteritis for further management.

Upon admission, a comprehensive workup was initiated. Echocardiography and electrocardiogram, along with serial cardiac enzymes and troponin I testing, supported the diagnoses of acute myocardial infarction and acute heart failure. Management included supplemental oxygen, dual antiplatelet therapy with aspirin and clopidogrel, anticoagulation with nadroparin, and low-dose diuretics. Intravenous meropenem was administered for antimicrobial coverage.

On the day of admission (Day 1), the patient developed circulatory instability, with her blood pressure plummeting to a nadir of 80/40 mmHg, accompanied by cool and clammy extremities. A diagnosis of septic shock was made, prompting the initiation of vasoactive agents and intravenous hydrocortisone (200 mg daily for two days). Hemodynamic stability was achieved after two days, allowing for the vasopressors to be successfully weaned. However, in the early morning (01:30) on Day 3, shortly thereafter, the patient experienced a sudden hypertensive crisis, with her blood pressure surging to 220/110 mmHg, accompanied by palpitations, headache, profuse sweating, and facial flushing. These symptoms gradually abated following the administration of oral captopril. Similar paroxysmal episodes recurred throughout her hospitalization, occasionally resolving spontaneously. Given this presentation of paroxysmal severe hypertension accompanied by the classic triad of palpitations, headache, and diaphoresis, a left adrenal pheochromocytoma was strongly suspected.

Subsequent targeted laboratory investigations revealed profoundly elevated catecholamine levels. Plasma catecholamines testing demonstrated dopamine at 9,533.4 pmol/L (reference range: ≤195.7 pmol/L), epinephrine at 10,199.6 pmol/L (reference range: ≤709 pmol/L), and norepinephrine at 36,160 pmol/L (reference range: 414-4,435.5 pmol/L). Twenty-four-hour urinary catecholamines showed epinephrine at 1,900.96 nmol/24 hours (reference range: 4.31-61.6 nmol/24 hours), norepinephrine at 3,071.67 nmol/24 hours (reference range: 60-352 nmol/24 hours), and dopamine at 4,755.46 nmol/24 hours (reference range: 750-2,088 nmol/24 hours). Other relevant tests included 24-hour urinary vanillylmandelic acid (VMA) at 11.79 mg/24 hours (reference range: 0-12 mg/24 hours).

A secondary hypertension panel was performed to investigate other potential causes of hypertension and adrenal dysfunction, which returned results within normal ranges, thereby helping to exclude conditions such as primary aldosteronism or Cushing's syndrome: angiotensin II: 34.50 pg/mL, cortisol: 182 ng/mL, adrenocorticotropic hormone: 28.90 pg/mL, aldosterone: 98.80 pg/mL, renin activity: 41.60 µIU/mL, and aldosterone-to-renin ratio (ARR): 2.38.

The diagnosis was confirmed based on Endocrine Society guidelines, which require both (1) unequivocally elevated biochemical evidence of catecholamine excess and (2) confirmatory imaging to localize the tumor [2].

Our patient met both criteria with massively elevated plasma/urinary catecholamines and a corresponding adrenal mass on CT. Histopathological confirmation is ultimately obtained after surgical resection.

**Figure 1:**
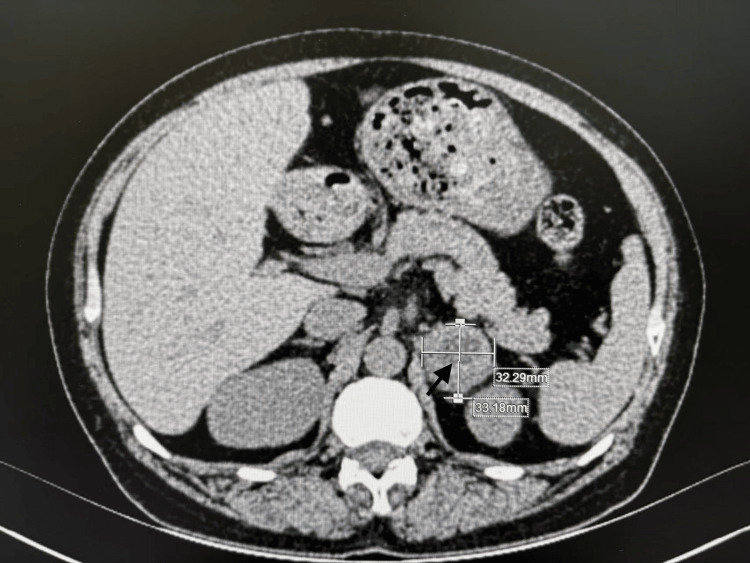
Abdominal CT scan showing a mixed-attenuation left adrenal mass (indicated by the arrow), measuring approximately 3.3 × 3.2 cm CT: computed tomography

An abdominal CT scan confirmed a mixed-attenuation mass in the left adrenal gland, measuring approximately 3.3 × 3.2 cm. Notably, coronary CT angiography (CTA) revealed no evidence of coronary artery stenosis (Figure 1).

Following diagnosis, the patient was started on phenoxybenzamine for alpha-adrenergic blockade as preoperative preparation. The transition from septic shock to paroxysmal hypertensive crises with the classic triad was the pivotal diagnostic clue that shifted suspicion from a purely infectious etiology to an underlying pheochromocytoma.

**Table 1:** Key laboratory findings at admission and during diagnostic workup for pheochromocytoma Elevated plasma and urinary catecholamine levels are the key biochemical findings confirming the diagnosis of pheochromocytoma. WBC: white blood cell count, CRP: C-reactive protein, PCT: procalcitonin, BNP: B-type natriuretic peptide, VMA: vanillylmandelic acid, ACTH: adrenocorticotropic hormone, ARR: aldosterone-to-renin ratio

Test category	Parameter	Patient value	Reference range	Unit
Inflammatory markers	WBC	15.98	3.5-9.5	×10⁹/L
Inflammatory markers	CRP	159.46	0-8	mg/L
Inflammatory markers	PCT	22.1	0-0.5	ng/mL
Cardiac biomarkers	Troponin I	1.36	0-0.04	ng/mL
Cardiac biomarkers	BNP	2,154	0-100	pg/mL
Plasma catecholamines	Dopamine	9,533.4	≤195.7	pmol/L
Plasma catecholamines	Epinephrine	10,199.6	≤709	pmol/L
Plasma catecholamines	Norepinephrine	36,160	414-4,435.5	pmol/L
24-hour urinary catecholamines	Epinephrine	1,900.96	4.31-61.6	nmol/24 hours
24-hour urinary catecholamines	Norepinephrine	3,071.67	60-352	nmol/24 hours
24-hour urinary catecholamines	Dopamine	4,755.46	750-2,088	nmol/24 hours
Other relevant tests	24-hour urinary VMA	11.79	0-12	mg/24 hours
Secondary hypertension panel	Angiotensin II	34.5	-	pg/mL
Secondary hypertension panel	Cortisol	182	-	ng/mL
Secondary hypertension panel	ACTH	28.9	-	pg/mL
Secondary hypertension panel	Aldosterone	98.8	-	pg/mL
Secondary hypertension panel	Renin activity	41.6	-	µIU/mL
Calculated ratio	ARR	2.38	-	-

## Discussion

Pheochromocytomas are rare neuroendocrine tumors, predominantly diagnosed in adults aged 30-50 years [3]. These tumors arise from chromaffin cells and are characterized by their potential to secrete catecholamines, leading to a wide spectrum of symptoms and potentially life-threatening complications if left undiagnosed [4]. Despite this rising prevalence, clinical recognition remains challenging, as evidenced by the review by Sutton et al. of 54 autopsy-proven cases where the diagnosis was missed antemortem [5].

The clinical presentation is highly heterogeneous, often featuring sustained or paroxysmal hypertension, severe headaches, palpitations, and sweating [6]. The frequency of paroxysms is unpredictable and varies from 30 times a day to a single episode every 2-3 months. Approximately 75% of patients have one or more spells per week. Duration ranges from a few minutes (usually 15-60 minutes) to days [7]. The profound cardiovascular impact of catecholamine excess is well-documented. Catecholamine-induced cardiomyopathy can result from direct myocardial toxicity, downregulation of beta-adrenergic receptors, and electrolyte disturbances, leading to myocardial cell damage [8,9]. Excessive adrenergic stimulation induces coronary vasoconstriction and vasospasm, explaining the significant troponin I elevation observed in our patient [10]. In severe cases, this progression culminates in pheochromocytoma-induced cardiogenic shock (PICS), characterized by acute left ventricular failure, reduced ejection fraction, pulmonary edema, elevated cardiac biomarkers, and electrocardiographic abnormalities [11]. Beyond direct cardiovascular effects, catecholamines trigger reactive oxygen species generation, pro-inflammatory cytokine release (including interleukin (IL)-1, IL-6, IL-8, tumor necrosis factor-alpha​​​​​​​ (TNF-α), and interferon-gamma​​​​​​​ (IFN-γ)), and subsequent endothelial injury [12,13]. This pathophysiological cascade shares mechanisms with stress cardiomyopathy (Takotsubo cardiomyopathy), which frequently accompanies pheochromocytoma crises [14]. The association between catecholamine excess and psychological stressors in triggering Takotsubo cardiomyopathy is well-documented [15,16], with recent evidence indicating increased incidence following COVID-19 infection due to inflammatory states, cytokine storms, and sympathetic activation [13].

In this case, the initial hypotension and elevated inflammatory markers (WBC, CRP, and PCT) were attributable to septic shock. However, the subsequent extreme hypertensive crises, the classic symptom triad, and the massively elevated catecholamines were pathognomonic for the pheochromocytoma crisis. The myocardial injury likely resulted from the combined effects of septic shock and catecholamine-induced cardiotoxicity.

The initial presentation with hypotension, dizziness, and shock in the context of an adrenal mass rightly raises the differential of an Addisonian crisis (adrenal insufficiency). Several key features, however, argued against this and pointed toward a pheochromocytoma crisis. First, the patient's hypotension was followed by dramatic, paroxysmal hypertensive crises, which are characteristic of pheochromocytoma, but not seen in adrenal insufficiency. Second, the classic symptomatic triad of palpitations, headache, and profuse sweating during these paroxysms is highly specific for catecholamine excess. Third, while hydrocortisone was administered for septic shock, the subsequent hypertensive crises occurred, which would be unexpected in a patient with cortisol deficiency. Biochemically, the diagnosis was confirmed by profoundly elevated plasma and urinary catecholamine levels. Although the adrenal mass was initially noted incidentally on a CT scan for abdominal symptoms, the definitive diagnosis of pheochromocytoma was driven by the subsequent classic clinical presentation and targeted biochemical confirmation. It is also important to note that beyond the cardiovascular manifestations described, a pheochromocytoma crisis can present with other features such as hyperthermia, altered mental status, and multi-organ failure.

Our case exemplifies a frequently overlooked trigger for such crises: common systemic infections. Previous reports have emphasized dramatic precipitants such as surgical procedures. Louis et al. described pheochromocytoma-induced cardiogenic shock (PICS) following thyroidectomy requiring venoarterial extracorporeal membrane oxygenation (VA-ECMO)​​​​​​​ support [9], while Guo et al. reported catastrophic cardiovascular collapse during anesthesia induction [10]. Similarly, Wang et al. identified pheochromocytoma as the underlying etiology in cases of acute decompensated heart failure and cardiogenic shock [17].

In contrast, our patient's pheochromocytoma crisis was precipitated by acute gastroenteritis and subsequent septic shock, common medical conditions rarely associated with such dramatic presentations. This unique trigger expands the clinical spectrum beyond the conventional perioperative setting. The initial septic shock obscured the underlying endocrinopathy, but the subsequent extreme blood pressure fluctuations and classic symptom triad provided crucial diagnostic clues. Early suspicion enabled targeted biochemical and imaging confirmation, while comprehensive conservative management addressing both infection and catecholamine excess achieved stabilization without requiring invasive mechanical support. In summary, this case demonstrates a sequential cascade: a common gastrointestinal infection precipitated septic shock, which acted as a powerful stressor to trigger a pheochromocytoma crisis, ultimately manifesting as acute myocardial infarction and cardiogenic shock.

## Conclusions

Pheochromocytoma-induced cardiogenic shock represents a rare but devastating clinical entity. This case highlights that common infections can be potent triggers for pheochromocytoma crises. Therefore, clinicians must maintain a high index of suspicion for this condition in any patient with unexplained, labile hemodynamics, regardless of a concurrent common illness. Early recognition, cessation of inappropriate vasopressors, and initiation of alpha-adrenergic blockade are critical steps in management. Timely diagnosis and aggressive, multimodal resuscitation are paramount for optimizing outcomes and avoiding the need for highly invasive support modalities.
